# Identification and Functional Implications of the E5 Oncogene Polymorphisms of Human Papillomavirus Type 16

**DOI:** 10.3390/tropicalmed9070140

**Published:** 2024-06-26

**Authors:** Antônio Humberto P. da Silva-Júnior, Ruany Cristyne de Oliveira Silva, Ana Pavla A. Diniz Gurgel, Marconi Rêgo Barros-Júnior, Kamylla Conceição Gomes Nascimento, Daffany Luana Santos, Lindomar J. Pena, Rita de Cássia Pereira Lima, Marcus Vinicius de Aragão Batista, Bárbara Simas Chagas, Antonio Carlos de Freitas

**Affiliations:** 1 Laboratory of Molecular Studies and Experimental Therapy (LEMTE), Department of Genetics, Federal University of Pernambuco, Recife 50670-901, Pernambuco, Brazil; antoniohumbertojr@yahoo.com.br (A.H.P.d.S.-J.); ruanycristyne@hotmail.com (R.C.d.O.S.); marconijrr@gmail.com (M.R.B.-J.); kamyllacgomes@gmail.com (K.C.G.N.); daffany.luana@ufpe.br (D.L.S.); ritajpbio@gmail.com (R.d.C.P.L.); babisimas@gmail.com (B.S.C.); 2 Department of Engineering and Environment, Federal University of Paraiba, João Pessoa 58033-455, Paraíba, Brazil; apavla@yahoo.com.br; 3 Laboratory of Virology and Experimental Therapy, Instituto Aggeu Magalhães (IAM), Oswaldo Cruz Foundation, Recife 50670-901, Pernambuco, Brazil; lindomar.pena@fiocruz.br; 4 Laboratory of Molecular Genetics and Biotechnology (GMBio), Department of Biology, Federal University of Sergipe, São Cristóvão 49107-230, Sergipe, Brazil

**Keywords:** HPV 16, E5 variants, functional analysis, phylogenetic relationships

## Abstract

The persistence of the human papillomavirus type 16 (HPV16) infection on the cervical epithelium contributes to the progression of cervical cancer. Studies have demonstrated that HPV16 genetic variants may be associated with different risks of developing cervical cancer. However, the E5 oncoprotein of HPV16, which is related to several cellular mechanisms in the initial phases of the infection and thus contributes to carcinogenesis, is still little studied. Here we investigate the HPV16 E5 oncogene variants to assess the effects of different mutations on the biological function of the E5 protein. We detected and analyzed the HPV16 E5 oncogene polymorphisms and their phylogenetic relationships. After that, we proposed a tertiary structure analysis of the protein variants, preferential codon usage, and functional activity of the HPV16 E5 protein. Intra-type variants were grouped in the lineages A and D using in silico analysis. The mutations in E5 were located in the T-cell epitopes region. We therefore analyzed the interference of the HPV16 E5 protein in the NF-kB pathway. Our results showed that the variants HPV16E5_49PE and HPV16E5_85PE did not increase the potential of the pathway activation capacity. This study provides additional knowledge about the mechanisms of dispersion of the HPV16 E5 variants, providing evidence that these variants may be relevant to the modulation of the NF-κB signaling pathway.

## 1. Introduction

Infection caused by human papillomavirus (HPV) is a determining factor in developing cervical cancer, which is considered one of the leading causes of women’s deaths worldwide [1]. Virus-mediated immune escape contributes to a persistent infectious process in the cervical epithelium, which can progress to the establishment of cervical cancer [2,3,4].

One of the viral types with broad geographic distribution worldwide is HPV16 [5], which is also present in northeastern Brazil [6,7]. Phylogenetically, HPV16 is clustered within the Alpha-9 species group [8]. Members of this family are the most prevalent high-risk type associated with cervical cancer cases. Thus, some studies have focused on identifying the distribution of HPV16 genetic variants and discussing their roles in infection and cervical cancer development [9,10]. Chagas et al. [6] and Gurgel et al. [7] showed the distribution of HPV16 LCR, E6, E7, and L1 genetic variants in northeastern Brazil. The functions of the E6 and E7 oncogenes have been well-studied, and their contribution to malignancy well-characterized [10,11].

To date, HPV16 has been classified into four variant lineages: lineage A [European (E), Asian, E(As)]; lineage B [African-1 (Afr1a), and African-1 (Afr1b)]; lineage C [African-2 (Afr2a)]; and lineage D [North American 1 (NA1), Asian American 1 (AA1), Asian American 2 (AA2)] [8]. These groups shared over 95% similarity based on the LCR region [8,9,10,11,12,13,14]. However, this classification should be based on other regions of the genome, such as the L1, E6, and E7 genes, as well as the usage of the entire genome [8,15,16,17].

Another candidate for initiating the infection process in the cervical epithelium is the E5 oncogene. The HPV16 E5 oncoprotein is related to several cellular mechanisms in the initial phases of infection, thus contributing to carcinogenesis. However, its exact function in these processes and viral pathogenesis is still unclear [18]. Structurally, the HPV16 E5 protein is hydrophobic, with low molecular weight, and can be found in biological membranes such as the endoplasmic reticulum (ER), the Golgi apparatus (GA), and the plasmatic and nuclear membranes [18,19].

Regarding its biological role, the HPV16 E5 oncoprotein acts by deregulating multiple cellular mediators, such as the EGF growth factor and its receptors (EGFR) [19]. It can act on apoptosis inhibition, induced by the tumor necrosis factor ligand (TNFL), and modular genes related to cell motility and adhesion [20]. In the immunological system, the HPV16 E5 oncoprotein acts to modulate innate and adaptive responses to inflammatory processes and cytokine synthesis. It is also able to activate the nuclear factor kappa B (NF-κB) and diminish the molecular expression of classes I and II of the major histocompatibility complex (MHC) [2,18,20]. Studies have shown that the HPV16 E5 oncoprotein acts on the down-regulation of MHC-I, binding with a heavy chain and preventing its expression on the cell surface, avoiding detection of infected cells and its elimination; and blocking MHC-II molecules, reducing the immune response [21,22].

The E5 potentiates carcinogenesis directed by HPV16 when it works in conjunction with E6 and E7 oncoproteins. It also interferes with the regulation of important molecules, such as the EGFR and other molecules involved in the control of cell differentiation, survival, and growth, for instance, Bcl-2, Bax, Fas, and calnexin [19,20,21,23].

Therefore, this study aimed to investigate the E5 genetic variants of HPV16 to identify the possible effects of their polymorphisms on the infection process by regulating the NF-kB pathway.

## 2. Materials and Methods

### 2.1. Sample Characteristics

The methodology and the cohort used in this study were based on a previous study performed by our research group [24]. Clinical samples were collected from patients treated at the gynecology service of the Hospital das Clínicas of the Federal University of Pernambuco. In this study, 107 samples were obtained from patients aged between 18 and 82 years old. 

The collected material was stored in phosphate-buffered saline (PBS) and stored at −20 °C. For DNA extraction, the Genomic Prep Blood DNA kit (Amersham Bioscience, Amersham, UK) was used, following the manufacturer’s instructions. DNA quantification was performed using the NanoDrop spectrophotometer (Thermo Fisher Scientific, Waltham, MA, USA).

All study participants signed the free and informed consent form. The exclusion criteria were patients with immunodeficiency or who refused to sign the informed consent form. This study was approved by the ethics committee of the Federal University of Pernambuco (CAAE: 3606212.7.0000.5208).

### 2.2. PCR Amplification and Sequencing 

The quality and integrity of the extracted DNA were analyzed through amplification of the WAF-1 gene (endogenous control) [25]. To detect HPV DNA in the samples, the primers MY09/MY11 [26] were used. Then, a type-specific PCR was performed for the HPV16 E6 oncogene amplification [6]. Finally, for the amplification of the HPV16 E5 oncogene, the primers HPV16 E5 F 5′-GCATTGGACAGGACATAATG-3′ and HPV16 E5 R 5′-AGAACGTTTGTGTCGCATTG-3′ were used.

Sequencing of the HPV16 positive samples was carried out twice using the dideoxy-terminal fluorescent method, using ABI PRISM BigDye^®^ Terminator Cycle Sequencing V3.1 kit Ready Reaction (Thermo Fisher Scientific, Waltham, MA, USA).

### 2.3. Sequence Analysis

To analyze the nucleotide sequence, the Gap4 (version 4.0) and Prepag4 (version 1.5) programs from the Staden package [27] were used. After obtaining the sequences and analyzing their quality (only sequences with Phred values above 30 were used), the obtained sequences were compared with the reference sequence of the HPV16 A1 (K02718) lineage, using the Basic Local Alignment Search Tool (BLAST) [28]. HPV sequences deposited at the *Papillomavirus Episteme* database (PaVE) were also used to assess HPV16 E5 oncogene sequences’ variability. The analysis of polymorphic sites was carried out through multiple sequence alignments, using the CLUSTALW algorithm, from the MEGA6 software [29]. BDGP Splice Site Prediction tool was used to determine if the polymorphisms would be predicted in a splice site (https://fruitfly.org/seq_tools/splice.html (accessed on 12 June 2024)).

### 2.4. Epitope Prediction

The epitopes were predicted using the online server Immune Epitope Database and Analysis Resource (IEDB) (http://www.iedb.org/ (accessed on 23 May 2023)). For the MHC-I, the set of alleles most frequently found in the global population was used. In this context, the length of the epitope sequence (that varied between 9 and 10 mer), percentile rank <1, and the immunogenicity score >0.5 were considered. For class II MHC molecules, the criteria considered were the percentile value and the IC50 value (<50 nM).

### 2.5. Phylogenetic Analysis

The phylogenetic analysis was conducted using the neighbor-joining method with the Kimura two-parameters evolutionary model using the MEGA6 program [29]. For the support of the branches, 1000 non-parametric bootstrap replicates were used. Four clusters were identified as lineages A, B, C, and D, and the sublineages were identified as A1 (K02718), A2 (AF536179), A3 (HQ644236), A4 (AF534061), B1 (AF536180), B2 (HQ644298), C (AF472509), D1 (HQ644257), D2 (AY686579), and D3 (AF402678).

### 2.6. Selection Pressure Analysis

Selection pressure was performed by calculating the parameters of six codon substitution models, M0, M1, M2, M3, M7, and M8 using PAML [30]. To evaluate the best model that fits the data, the likelihood ratio test (LRT) was carried out.

### 2.7. Three-Dimensional Structure Prediction

Initially, to predict the tertiary structure of the HPV16 E5 oncoprotein, its reference sequence was obtained (AAA46938) from the NCBI Protein Database. As there was no template structure available in the Protein Data Base (PDB) for homology modeling, the modeling of the tertiary structure of HPV16 E5 was performed with the ab initio method, using QUARK [31] and Robetta [32] servers.

The structural model was predicted for each sample. Variations in the amino acid sequence were observed and ten different models were obtained for each sample. The models were refined through energy minimization using ModRefiner [33] and evaluated with PROCHECK [34], by using the stereochemical quality of the models in the Ramachandran plot, Anolea [35], Gromos [36], QMEAN6 [37], DFire [38], DSSP [39], and Promotif [40]. After analyzing the parameters, the best model was chosen to represent the protein structure. To predict the effect of polymorphic sites on the stability of the HPV16 E5 oncoprotein, the Site-Directed Mutator (SDM) server was used [41].

### 2.8. Codon Usage Analysis

The Graphical Codon Usage Analyzer online software, available at http://gcua.schoedl.de/ (accessed on 10 June 2023), was used to identify the preferential use of synonymous codons, rare or frequent, used by the HPV16 E5 variants, compared to those used by their mammalian host.

### 2.9. Plasmid Constructs

The chosen variants of the E5 oncogene (252 bp) with greater epidemiological relevance, together with its prototype, followed the same cloning and subcloning methodology described in [24].

### 2.10. Isolation of DNA from Recombinant Vectors, Cultivation, and Cell Transfection

After confirming the results through sequencing, the DNA of the recombinant vectors (containing the variant sequences of the HPV16 E5 gene and the prototype) were isolated through maxi-preparation using the Plasmid Plus Maxi Kit (Qiagen, Hilden, Germany). The maxi-preparation products were quantified and subsequently used in transfection assays. The human embryonic kidney (HEK-293) cell was used because it has been well established in the literature for co-transfection, using plasmids containing genes of HPV [24,42,43,44,45]. The cell cultivation and transfection steps followed the methodology described in [24].

### 2.11. Evaluation of Gene Expression through Luminescence

Cells that were co-transfected with the pcDNA3.1 (+) plasmid containing 1.5 µg of the E5 variant, the prototype, or with the empty pcDNA3.1 (+) vector (negative control). The (kB)3-Luc plasmid (1 ug) and a plasmid expressing Renilla luciferase (1 ng) as luminescence normalizer were subjected to the analysis of the activity of the NF-kB pathway, through the luminescence emitted and measured using the GloMax Microplate Luminometer^®^ (Promega, Madison, WI, USA) using the luciferase reporter assay system (Promega, Madison, WI, USA). To ensure comparable transfection efficiencies, results were normalized via renilla firefly along with the protein quantification.

### 2.12. Statistical Analysis

Statistical analysis was performed using one-way ANOVA followed by Bonferroni correction, where values of *p* < 0.05 were considered significant.

## 3. Results

### 3.1. Clinical Samples

After analyzing 107 positive HPV samples, a total of 24 samples were genotyped as HPV16, and variants of the E5 oncogene were observed in 19 samples (79.16%). Only positive HPV16 samples with a PHRED quality score ≥30 were eligible. Samples that presented low scores and inaccurate results were excluded.

The selected samples were designated as HPV16E5_06PE, HPV16E5_11PE, HPV16E5_14PE, HPV16E5_16PE, HPV16E5_25PE, HPV16E5_30PE, HPV16E5_33PE, HPV16E5_35PE, HPV16E5_45PE, HPV16E5_49PE, HPV16E5_55PE, HPV16E5_70PE, HPV16E5_71PE, HPV16E5_76PE, HPV16E5_78PE, HPV16E5_79PE, HPV16E5_85PE, HPV16E5_91PE, and HPV16E5_93PE.

### 3.2. Identification of Polymorphic Sites

After identifying and sequencing the positive HPV16 clinical samples, these data were compared with the K02718 reference sequence, which was deposited in the National Center for Biotechnology Information (NCBI) database. A total of seven polymorphisms in eleven clinical samples were identified, from which four were described as being non-synonymous substitutions (A3979C; T3988A; C3991G; and A4042G) and three as synonymous substitutions (C3991T; G4017A; and T4089C). At position 3991, the most common substitution was C3991G. However, the sample HPV16E5_49PE presented a different polymorphic site in this position, characterized as C3991T (Table 1). No instances of polymorphisms were found in splice sites.

The majority of the polymorphic samples were from HPV16E5_49PE (six substitutions), HPV16E5_55PE (five substitutions), HPV16E5_70PE (five substitutions), HPV16E5_85PE (five substitutions), HPV16E5_93PE (five substitutions), HPV16E5_14PE (four substitutions), HPV16E5_16PE (four substitutions), and HPV16E5_78PE (four substitutions). The greatest numbers of nucleotide substitutions were observed at positions 3979, 3991, 4017, and 4042 (Table 1). The mutations A3979C, T3988A, C3991T, and A4042G (Table 1) resulted in the amino acid substitutions I44L, L47I, L48V, and I65V, respectively.

### 3.3. T-Cell Epitope Prediction

Regarding the T-cell epitope prediction, the IEDB online platform was used to verify whether the I44L, L47I, L48V, and I65V mutations were located in regions of immunogenic epitopes for the MHC class I and II alleles.

The I44L amino acid change was identified in a site belonging to the T-cell epitope with MHC I binding activity (E5 38-46 ILLVLVLWI) and MHC II binding activity (E5 41-47 LVLWITAAS); the L47I change was found in a site belonging to the T-cell epitope with MHC I binding activity (E5 45-53 WITAASAFR); the L48V change was identified in a site belonging to the T-cell epitope with MHC I binding activity (E5 50-58 SAFRCFIVY) and MHC II binding activity (E5 51-59 FRCFIVYIV); the I65V change was identified in a site belonging to the T-cell epitope with MHC I binding activity (E5 60-68 VFVYIPLFL) and MHC II binding activity (E5 60-68 FUYIPLFLI).

The impact of the amino acid changes on the predicted epitopes was measured by comparing the prediction scores. The I44L change presented a slight decrease in the epitope prediction score (from 0.051153 to 0.047742), L47I and L48V presented significant increases in the epitope prediction scores (from 0.153919 to 0.561362 and from 0.452043 to 0.555289, respectively), and the I65V change presented a slight increase in the epitope prediction score (from 0.401191 to 0.418311). Although the amino acid changes presented a significant impact on the epitope prediction score, the non-synonymous substitutions were conservative changes (aliphatic hydrophobic amino acids), which suggests that the effects on protein function might be limited.

### 3.4. Phylogenetic Analysis

**Figure 1:**
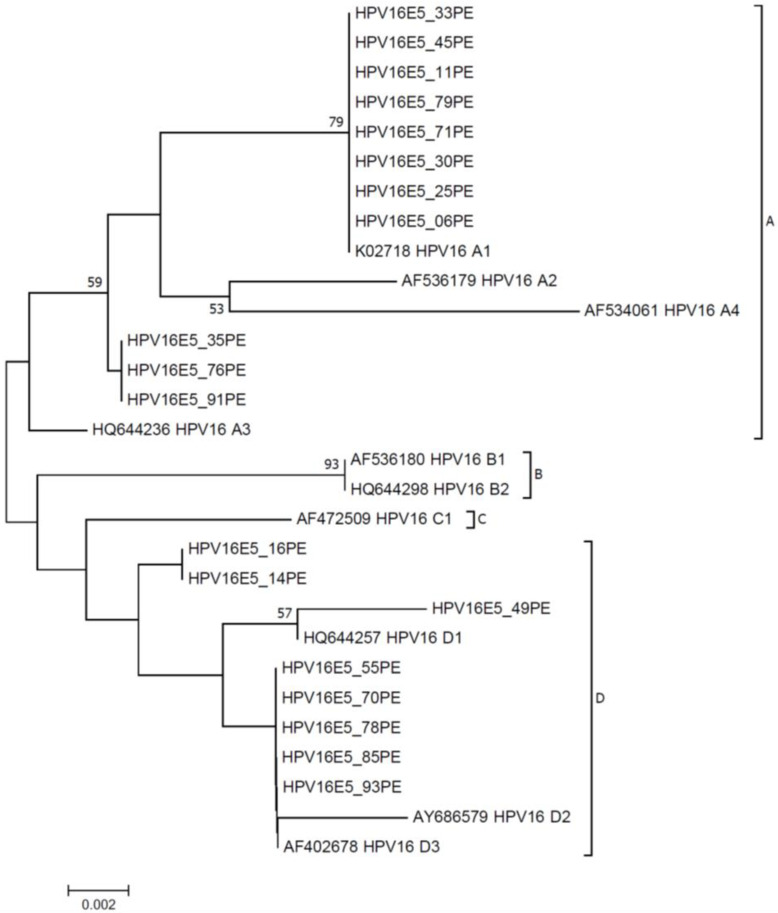
The neighbor-joining phylogenetic tree is based on the HPV16 E5 gene sequences. Clusters representing lineages A, B, C, and D are shown. A bootstrap of 1000 replicates determined branch support. Only bootstrap values higher than 50% are shown. Scale bar represents nucleotide substitutions per site.

The phylogenetic tree was constructed by comparing the E5 sequences. The results showed that the 19 analyzed sequences presented a grouping profile characteristic of the four HPV16 lineages (A, B, C, and D). Among them, eleven were classified as belonging to lineage A, and eight to lineage D (Figure 1). The tree presents groups that allowed the classification of the isolates in lineages. It was observed that the isolates HPV16E5_06PE, HPV16E5_11PE, HPV16E5_25PE, HPV16E5_30PE, HPV16E5_33PE, HPV16E5_45PE, HPV16E5_71PE, and HPV16E5_79PE were grouped in the A1 sublineage branch. The isolates HPV16E5_35PE, HPV16E5_76PE, and HPV16E5_71PE were classified as lineage A. The isolates HPV16E5_16PE, HPV16E5_14PE, HPV16E5_49PE, HPV16E5_55PE, HPV16E5_70PE, HPV16E5_78PE, HPV16E5_85PE, and HPV16E5_93PE were classified as lineage D (Figure 1). It is important to point out that the most polymorphic E5 samples clustered together in lineage D.

### 3.5. Determination of the Selection Pressure

The HPV16 E5 protein presented ω values between 0 and 1, which means that in this population, the E5 protein is under selection. The LRT test estimated that the codon substitution models that most fit the data were M2 and M3, with ω = 0.91480 (Table 2).

The E5 protein of HPV16 presented globally, purifying or negative selection. However, the possibility of specific polymorphic sites being or not under diversifying or positive selection pressure was checked. The analysis showed that the amino acid residues 48L (*p* = 0.037; 10.141 ± 0.857) and 65I (*p* = 0.007; 10.184 ± 0.570) were under diversifying selection, according to the M2, M3, and M8 models, and was statistically significant according to the LRT test. The 44I (*p* = 0.495; 5.494 ± 4.766) and 47L (*p* = 0.493; 5.514 ± 4.766) residues were under positive selection according to the M8 model, but it was not statistically significant.

### 3.6. Structural Analysis of the HPV16 E5 Protein

**Figure 2:**
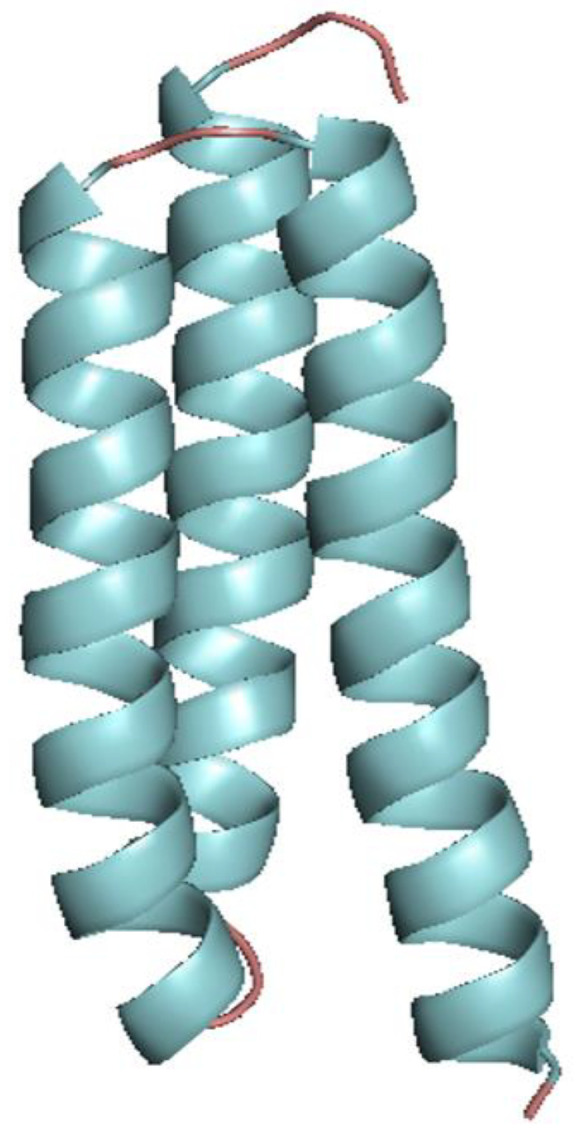
Three-dimensional model of the HPV16 E5 protein determined by ab initio method. The structure shows the three alpha-helix domains.

Many polymorphisms identified in the HPV16 E5 variants are located at the transmembrane helix regions, as can be observed in the tertiary structure of variants HPV16E5_14PE, HPV16E5_16PE, HPV16E5_49PE, HPV16E5_55PE, HPV16E5_70PE, HPV16E5_78PE, HPV16E5_85PE, and HPV16E5_93PE (Figure 2).

Because the variants HPV16E5_49PE and HPV16E5_14PE presented all non-synonym mutations identified in the study, they were the only variants compared with the reference protein structure. The best model of the reference HPV16 E5 protein presented 98.7%, the variant HPV16E5_14PE presented 91.9%, and the variant HPV16E5_49PE presented 93.2% of amino acid residues in the most favored regions in the Ramachandran plot. These results show that it was possible to obtain a good-quality model for the mutations’ structural evaluation.

The E5 protein of HPV16 is a small hydrophobic protein of 83 amino acids, which can form polar interactions, establishing its transmembrane domains. In this E5 protein topological model, we found hydrophobic amino acid residues, especially serine, threonine, and alanine residues, making the transmembrane alpha-helices regions (Figure 2).

**Figure 3:**
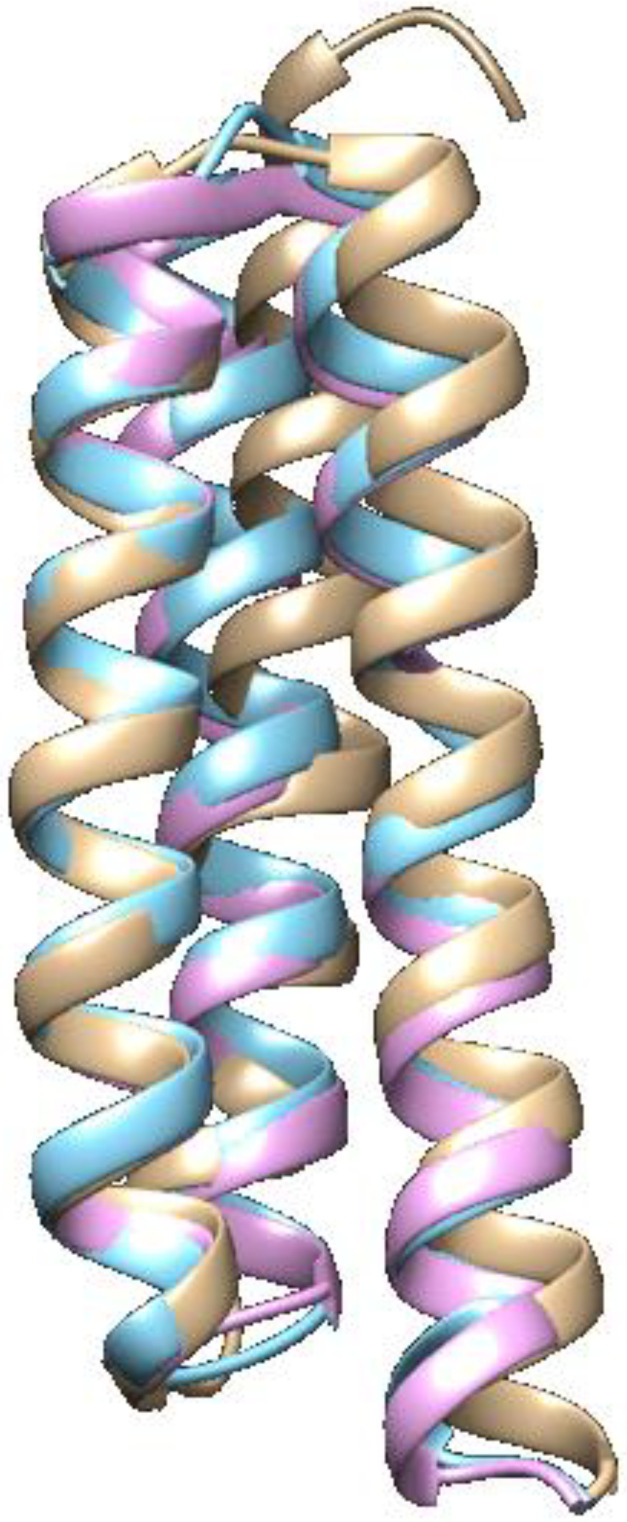
Structural alignment of the reference HPV16 E5 oncoprotein (brown), the variant HPV16E5_14PE (blue), and the variant HPV16E5_49PE (pink).

The non-synonymous mutations are located in alpha-helix regions. Despite the observed mutations, the alignment of the E5 reference protein structures with the two variants shows that there were no significant modifications to the protein structure (Figure 3).

Evaluating the impact of these mutations on protein stability, we observed that the I44L mutation presented the predicted value of ΔΔG as 0.37, which causes a small increase in protein stability. The mutations L47I, L48V, and I65V presented negative values of ΔΔG, indicating that these mutations are associated with reduced stability of the E5 protein (Table 3).

### 3.7. Genetic Variability and Preferential Codon Usage

The non-synonymous mutations I44L, L47I, L48V, and I65V were analyzed to ensure whether the codon changes in these positions reflected a preferential codon usage correlated with *Homo sapiens* (Table 4). The obtained results did not correlate with the codon usage and in regions with non-synonymous substitutions, which suggests that they do not have adaptive advantages in this virus-host relationship (Supplementary Figure S1).

### 3.8. Functional Activity of HPV16 E5 Protein Variants

The analysis of the E5-HPV16 oncogene variants was carried out through luciferase assays using the NF-κB pathway as a model of functionality. The high-risk HPV E5 oncoprotein can activate the NF-κB pathway [46,47]. The pathway is important for immune functions, but its excessive activation can lead to chronic inflammation [47].

**Figure 4:**
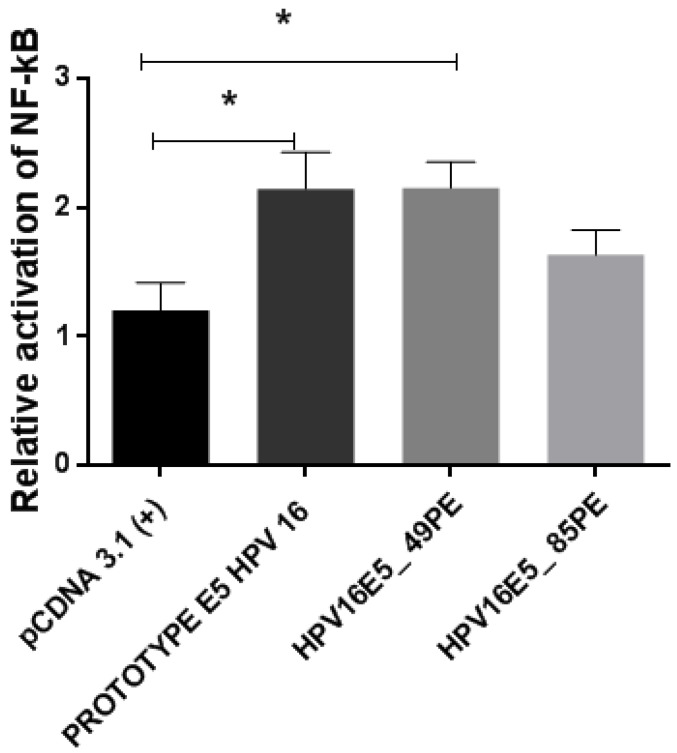
Effect of HPV16 E5 on NF-κB pathway activation in transfected HEK-293 cells. Each group was transfected with the pcDNA3.1 (+) plasmid containing the E5 variant, or the prototype (reference gene without any of the studied variations), or with the empty pcDNA3.1 (+) vector (negative control). Furthermore, all groups of cells were cotransfected with the (kB)3-Luc plasmid (1 ug) containing an NF-kB-dependent firefly luciferase reporter and a plasmid expressing Renilla luciferase as a luminescence normalizer. The bars represent the average of each condition performed in three experimental replicates in triplicates, with *p*-value = 0.02. * indicates *p*-value < 0.05.

Two HPV16 E5 variants (HPV16E5_49PE and HPV16E5_85PE) were used in this analysis, which belongs to the lineage D. The effects of variant strains on the NF-κB pathway were compared to those found in cells expressing the HPV16 E5 reference protein (which were transfected with the prototype) and cells transfected with pCDNA3.1 (+) (empty plasmid). Among the obtained results, it was observed that the E5 prototype can activate the NF-κB pathway when compared to the empty plasmid. The variant HPV16E5_49PE had the same potential to increase the NF-κB pathway activation capacity compared to the prototype. The variant HPV16E5_85PE activated the NF-κB pathway to a lesser extent when compared to the prototype (Figure 4). Comparing the mean values of the analyzed groups (pCDNA3.1 (+), prototype E5 HPV16, and variants) there was a significant difference with *p*-value = 0.02. When different pairwise comparisons were made, there was a significant difference between the prototype with pCDNA and pCDNA with the HPV16E5_49PE variant, with a *p*-value < 0.05.

## 4. Discussion

HPV16 is one of the most prevalent HPV types, with broad distribution in Brazil, especially in the northeast region [6,7,12]. Numerous HPV16 variants have been identified in regions and ethnic groups around the world. These variants could exhibit different oncogenic potentials, indicating that some specific lineages can affect the persistence of HPV infection and the progression of pre-cervical cancer lesions [48,49,50]. In this study, we determined the presence of HPV16 E5 variants and studied the possible biological effects of these variants. No novel variants were found, as all polymorphisms identified in this study population have been reported in previous studies [51,52]. However, the distribution and functional analysis of the HPV16 E5 oncogene variants in Brazil has not yet been evaluated, which motivated us to investigate the possible biological effects of HPV16 E5 variants that were identified from clinical samples.

The data obtained in this study provide information on the circulation of these variants in northeastern Brazil (this study being a pioneering study for the region). Another important aspect, which was investigated in a previous study [53], concerns the potential for immune evasion of these variants. In our results, we observed that some non-synonymous changes did not cause great changes in the architecture of the E5 oncoprotein. Therefore, it is, necessary to verify whether these changes will be capable of producing greater stability for HPV16 during the initial phases of the infectious cycle, influencing MHC class I and II molecules retention [54], blocking the cytotoxic T lymphocyte activation affecting the immunological response [55], and increasing the risk of developing cancer [56].

Regarding phylogenetic analysis, the results presented in this study pointed to the circulation of HPV16 E5 variants belonging to the lineages A (European lineage) and D (non-European lineage). Studies have shown that non-European lineages (B, C, and D) present increased carcinogenic potential when compared with the European lineage (A) [57,58,59].

Phylogenetically, through the molecular analysis of intra-type variations, we observed the formation of two very distinct branches, in which the isolates are distributed in lineages A and D. Studies comparing HPV16 strains show that non-European strains (B, C, and D) are more pathogenic when compared to European strains (A) [8]. Consistent results confirm a 2- to 4-fold increase in cervical neoplasia in the case of non-European strains [53,57,60]. Specifically, line D compared to line A has 4–35 times more association with adenocarcinoma [61,62]. Furthermore, we compared the rate of synonymous and non-synonymous substitutions, and the results showed that models M2 and M3 represent adequate models of codon substitution, with the HPV16 E5 oncogene in either a negative or purifying selection. We also verified that the specific site of the 48L and 65I amino acid residues was under a diversifying selection of the complete genome in twelve cervical HPV16 isolates. By analyzing the molecular evolution and selective pressure of the variants, we observed that the HPV16 E5 oncogene is under a positive selection. Our results showed a high frequency of I44L and I65V mutations in the northeastern Brazil population. The I44L polymorphism was predicted to be associated with increased protein stability, whereas I65V is related to reduced stability. In this context, polymorphisms were evaluated individually. However, the variants presented in our study have multiple polymorphisms, which could modify cell behavior. The construction of a combined I44L and I65V (Leu^44^Val^65^) variant was responsible for a decrease in the G0-G1 phase; on subsequent steps during the cell cycle, it was responsible for an increase in the G2-M phase, suggesting that this variant is related to cell growth [63]. However, I44L and I65V were studied as a combined variant [63], and the role of the individual changes is unclear. In our study, the I65V mutation had a negative ΔΔG, which was predicted to be associated with the protein malfunction. Therefore, these results suggest that these mutations may impact the E5 function, explaining the changes in the cell cycle behavior under the HPV16 infection.

HPV16 consists of two functional domains. The first domain is related to the voltage-gated motif, and the second to the 16kDa ATPase pump, which is associated with the acidifying endosome process [64,65]. The combination of I44L and I65V in the HPV16 E5 variant has been shown to lead to reduced p21 expression [63]. Whether the other two non-synonymous substitutions, L47I and L48V, affect p21 expression is unknown.

In addition to in silico studies, it is also important to evaluate these variants functionally. Therefore, we chose to analyze the E5 protein of HPV16 during the interaction and signaling with the NF-kB pathway. The NF-κB pathway corresponds to a family of transcriptional factors that bind to responsive κB sequences found in DNA. These factors are responsible for expressing genes related to the inflammatory response, proliferation, differentiation, adhesion, and apoptosis [66,67,68,69,70,71]. The exaggerated or even constitutive activation of the pathway has been detected in many human diseases [72].

The results obtained through the functional study were used to evaluate the activity of the NF-kB pathway mediated by the HPV16 E5 oncogene. These results show that the prototype can activate the NF-κB pathway when compared to the empty plasmid. The HPV16E5_49PE variant showed the same potential as the prototype, and the HPV16E5_85PE variant activated the pathway to a lesser extent when compared to the prototype. The expression of E5 leads to the activation of the NF-kB pathway [24,46]. In this case, the results show that wild-type E5 itself can activate the pathway together with the variants, even if the HPV16E5_85PE variant activated the pathway in a lower percentage (Figure 4). The other oncoproteins (E6 and E7) of high-risk HPVs also modulate the expression of genes responsive to NF-ĸB [73,74]. The HPV16 E7 protein interferes with NF-κB signaling, causing a reduction in its signaling [75]. In another study, even under stimulation of the TNF-α cytokine, the HPV16 E7 oncoprotein was less responsive, exhibiting attenuated NF-κB signaling [76]. A more recent study with the HPV31 E5 also looked at the NF-kB pathway. It was observed that all the HPV31 E5 variants used in the study increased the pathway compared to the prototype [24]. The NF-kB pathway has been considered an attractive therapeutic target for cancer treatment [72]. Its stimulation can increase metastatic potential, tumor angiogenesis, and cell proliferation, and can block apoptosis, thus leading to a greater risk of developing cancer.

Although this study provides relevant information on the impact of HPV16 E5 mutations on the NF-kB pathway, it is important to highlight that further studies are needed to assess what other potential biological mechanisms of E5 may be acting on the infected cell. These mechanisms are important for understanding the role of E5 in maintaining the HPV infection cycle and establishing early carcinogenesis. Understanding the mechanisms through which the HPV16 E5 mutations modulate vital cellular processes (such as proliferation, differentiation, apoptosis, survival, adhesion, migration, and invasion) is paramount for the identification of therapeutic targets and the development of drugs that can inhibit the transformation process associated with E5 [77]. In this context, the main limitation of our study is that it is difficult to pinpoint the effect of the amino acid changes as E5 is involved in multiple processes.

## 5. Conclusions

The HPV16 E5 oncogene is involved in multiple cellular processes, and the analysis of its biological behavior in the infectious process must be thorough. This study evaluated the structural variations in the E5 oncogene that resulted from mutations and provided additional knowledge about the mechanisms of dispersion of HPV16 E5 genetic variants found in northeastern Brazil. Furthermore, this study provided evidence that these variants may be relevant to the modulation of the NF-κB signaling pathway. Therefore, this study adds information about HPV infection profiles and provides important data to better understand how high-risk HPV genetic variants may be related to their clinical consequences.

## Figures and Tables

**Table 1:** Polymorphic sites of the HPV16 E5 variants. The data show the polymorphic site position of the analyzed sequences in comparison to the HPV16 K02718 reference sequence. Asterisk (*) indicates conservative sites.

	Position of Polymorphic Nucleotides at Oncogene HPV16 E5					
Samples	3979	3988	3991	4017	4042	4089
Reference K02718	A	T	C	G	A	T
HPV16E5sample_14PE	C	*	G	A	G	*
HPV16E5sample_16PE	C	*	G	A	G	*
HPV16E5sample_35PE	C	*	*	*	G	*
HPV16E5sample_49PE	C	A	T	A	G	C
HPV16E5sample_55PE	C	*	G	A	G	C
HPV16E5sample_70PE	C	*	G	A	G	C
HPV16E5sample_76PE	C	*	*	*	G	*
HPV16E5sample_78PE	C	*	G	A	G	*
HPV16E5sample_85PE	C	*	G	A	G	C
HPV16E5sample_91PE	C	*	*	*	G	*
HPV16E5sample_93PE	C	*	G	A	G	C

**Table 2:** Selective pressure estimates in HPV16 E5 protein based on maximum likelihood. The model that best fits the data is in bold. * indicates that the values were determined from the arithmetic mean.

ORF	Model	lnL	ω = dN/dS *	Parameters
E5	M0	−475.636423	0.81436	ω = 0.81436
	M1	−472.450423	0.33674	p_0_ = 0.66326 (p_1_ = 0.33674) ω_0_ = 0.00000; ω_1_ = 1.00000
	**M2**	**−466.217010**	**0.91480**	**p_0_ = 0.96934; p_1_ = 0.00000 (p_2_ = 0.03066) ω_0_ = 0.35685; ω_1_ = 1.00000; ω_2_ = 18.55892**
	**M3**	**−466.217010**	**0.91480**	**p_0_ = 0.04767; p_1_ = 0.92168 (p_2_ = 0.03066) ω_0_ = 0.35685; ω_1_ = 0.35685; ω_2_ = 18.55893**
	M7	−472.486769	0.30000	p = 0.00500; q = 0.01155
	M8	−466.218500	0.91560	p_0_ = 0.96934 (p_1_ = 0.03066) p = 55.05023; q = 99.00000; ω_s_ = 18.56975

**Table 3:** Effect of mutations on the structure of HPV16 E5 protein. Positive predicted ΔΔG values are related to increased protein stability. Negative predicted ΔΔG values are related to reduced protein stability.

Mutation	Wild Residue	Residue Position	Mutant Residue	Predicted ΔΔG	Outcome
1	I	44	L	0.37	Increased stability
2	L	47	I	−0.48	Reduced stability
3	L	48	V	−0.67	Reduced stability
4	I	65	V	−0.35	Reduced stability

**Table 4:** Analysis of polymorphic sites and changes in the sequence of preferential codon usage in HPV16 E5 oncogene variants. The table shows the change in the codons in the non-synonymous regions and the comparison with the reference sequence (K02718).

Isolate	Position of Non-Synonymous Polymorphisms	Codon Usage	Percentage of Use (%)	Relative Adaptiveness (%)
**K02718**	44	ATA	17	36
	47	TTA	8	20
	48	CTA	7	18
	65	ATA	17	36
**HPV16E5_14PE and HPV16E5_16PE**	44	CTA	7	18
	47	TTA	8	20
	48	GTA	12	26
	65	GTA	12	26
**HPV16E5_49PE**	44	CTA	7	18
	47	ATA	17	36
	48	TTA	8	20
	65	GTA	12	26
**HPV16E5_35PE, HPV16E5_76PE and HPV16E5_91PE**	44	CTA	7	18
	47	TTA	8	20
	48	CTA	7	18
	65	ATA	17	36
**HPV16E5_55PE, HPV16E5_70PE, HPV16E5_78PE, HPV16E5_85PE and HPV16E5_93PE**	44	CTA	7	18
	47	TTA	8	20
	48	GTA	12	26
	65	GTA	12	26

## Data Availability

All the relevant data are provided in the article.

## Supplementary Materials

The following supporting information can be downloaded at: https://www.mdpi.com/article/10.3390/tropicalmed9070140/s1, Figure S1: The graphs indicate how often the codons are used. The bars in red indicate a percentage less than 10%; the bars in gray indicate a percentage less than 20% of the use of preferential codons. A: Reference sample K02718; B: variants HPV16E5_14PE and HPV16E5_16PE; C: variant HPV16E5_49PE; D: variants HPV16E5_35PE, HPV16E5_76PE, and HPV16E5_91PE; E: variants HPV16E5_55PE, HPV16E5_70PE, HPV16E5_78PE, HPV16E5_85PE, and HPV16E5_93PE.
