# Supplementary material for: Identification and Functional Implications of the E5 Oncogene Polymorphisms of Human Papillomavirus Type 16
Source: Trop Med Infect Dis. 2024 Jun 26;9(7):140. doi: 10.3390/tropicalmed9070140 (PMC11281449; doi:10.3390/tropicalmed9070140)
Supplement: Supplementary file 1 [file tropicalmed-09-00140-s001.zip › tropicalmed-2973093-supplementary.pdf]

Supplementary Figure S1: The graphs indicate how often the codons are used. The bars in red indicate a percentage less than 10%; the bars in gray indicate a percentage less than 20% of the use of preferential codons. A: Reference sample K02718; B: variants HPV16E5_14PE and HPV16E5_16PE; C: variant HPV16E5_49PE; D: variants HPV16E5_35PE, HPV16E5_76PE and HPV16E5_91PE; E: variants HPV16E5_55PE, HPV16E5_70PE, HPV16E5_78PE, HPV16E5_85PE and HPV16E5_93PE.

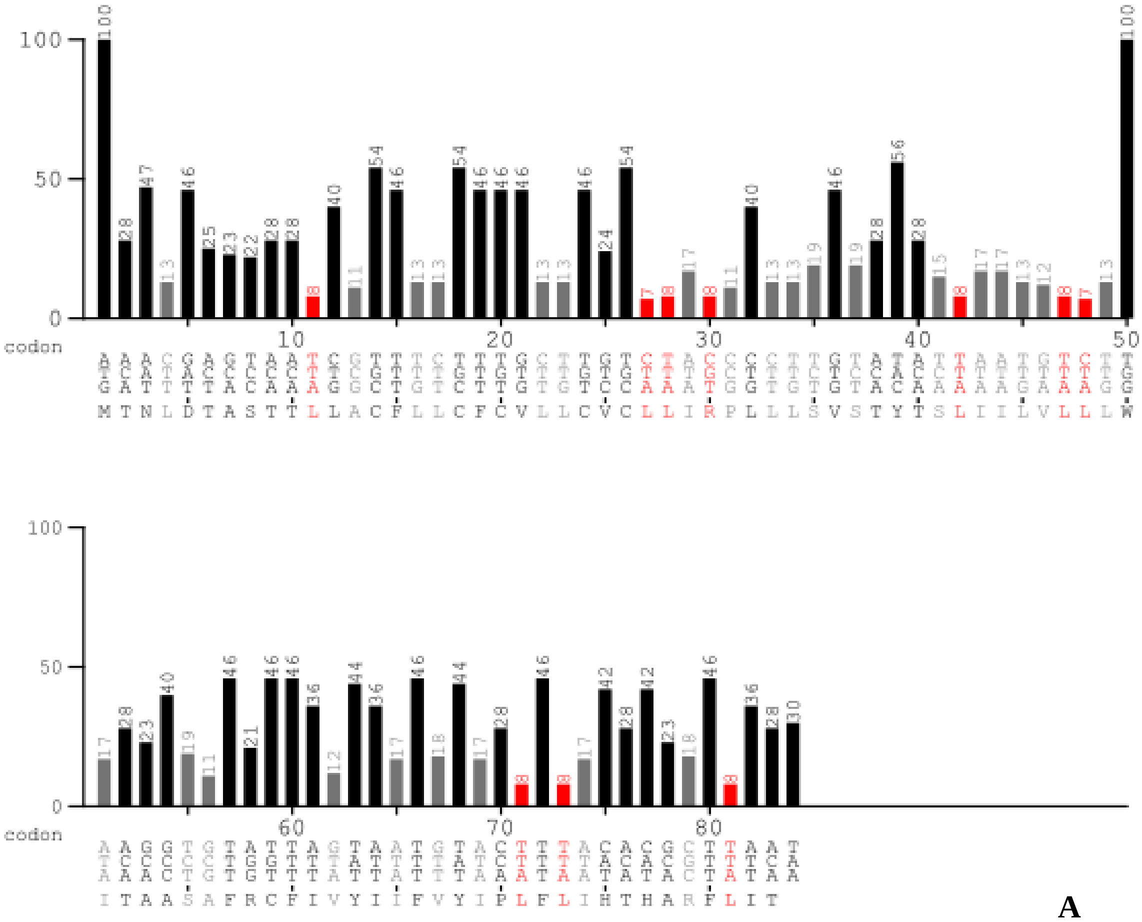

A

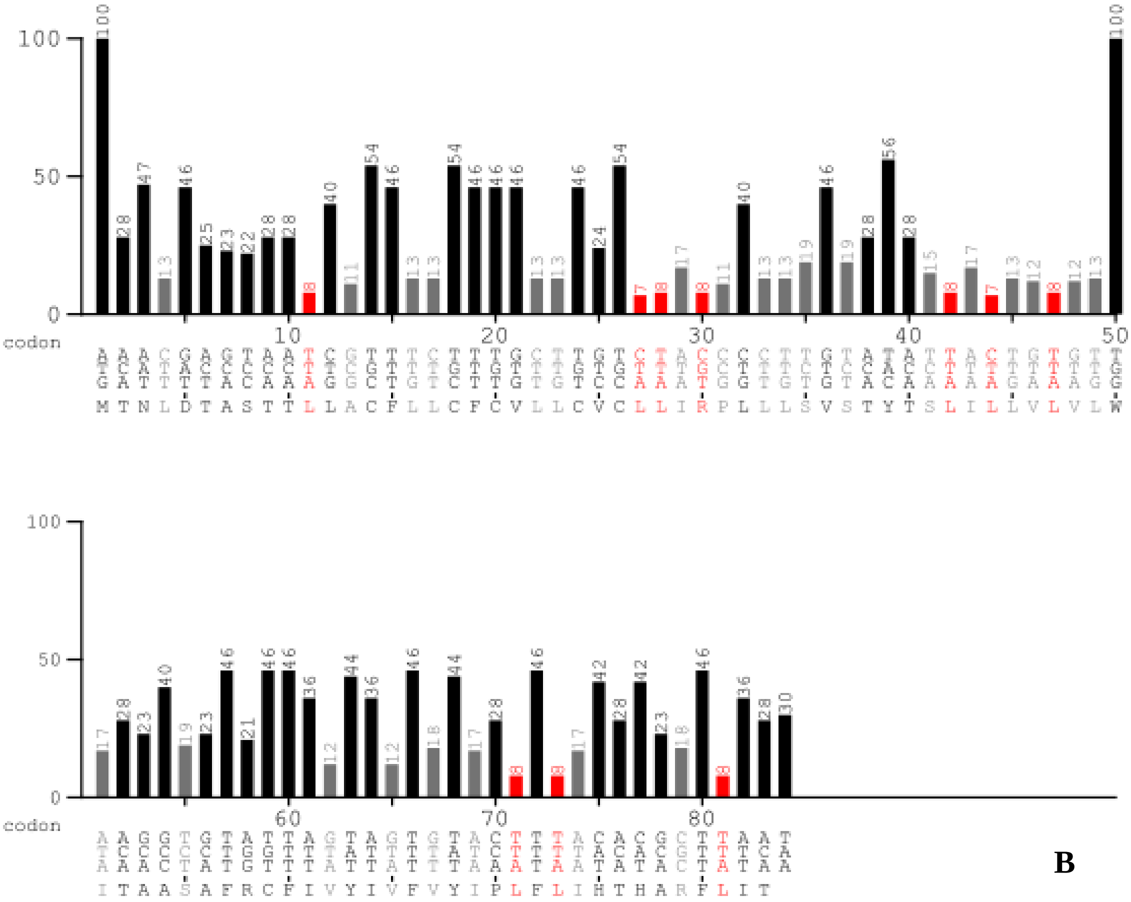

B

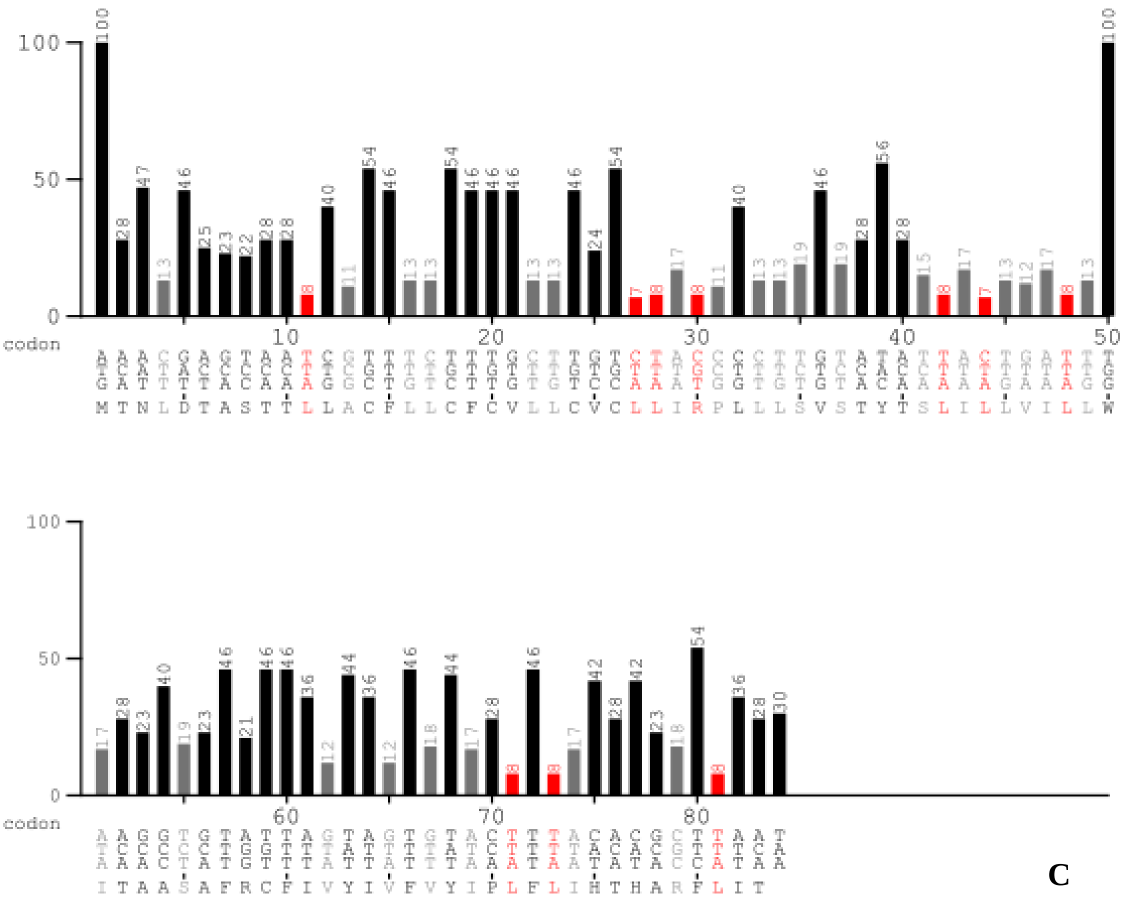

C

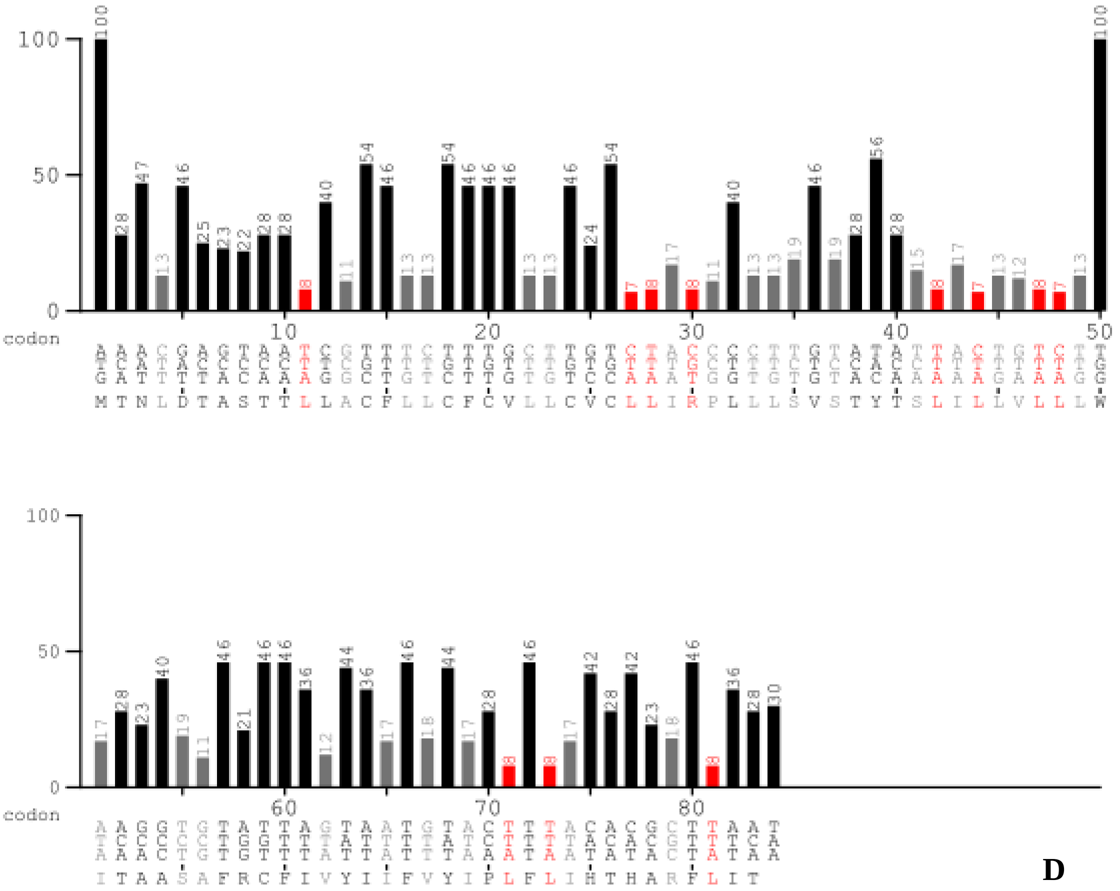

D

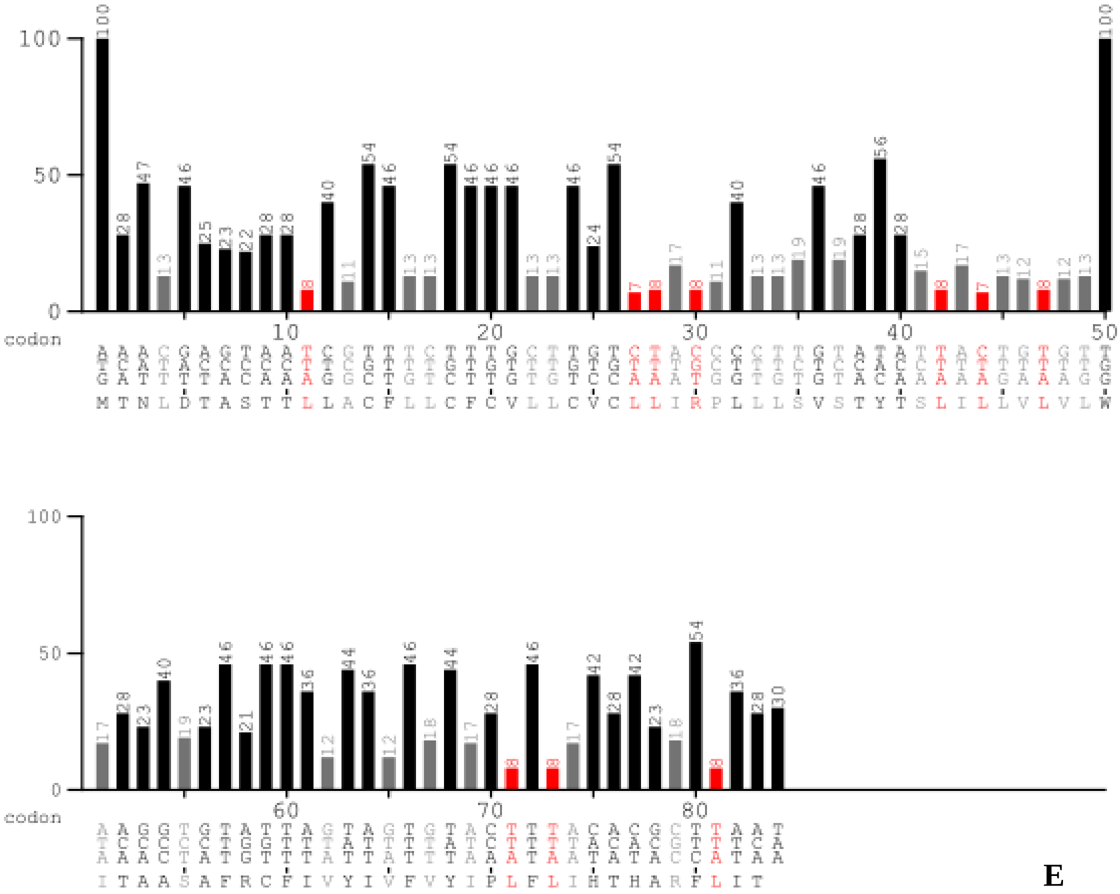

E
